# Tracing of Human Tumor Cell Lineages by Mitochondrial Mutations

**DOI:** 10.3389/fonc.2020.523860

**Published:** 2020-12-03

**Authors:** Paulo Refinetti, Stephan Morgenthaler, William G. Thilly, Christian Arstad, Per O. Ekstrøm

**Affiliations:** ^1^ Chair of Applied Statistics, Mathematics Section, School of Basic Sciences, École Polytechnique Fédéral de Lausanne (EPFL), Lausanne, Switzerland; ^2^ Department of Biological Engineering, Massachusetts Institute of Technology, Cambridge, MA, United States; ^3^ Department of Tumor Biology, Norwegian Radium Hospital, Oslo, Norway

**Keywords:** human, tumor, metastasis, lineage tracing, three dimension

## Abstract

**Background:**

Previous studies have shown the value in studying lineage tracing in slices of human tumors. However, a tumor is not a two-dimensional structure and to better understand how a tumor, and its corresponding metastasis grow, a three-dimensional (3-D) view is necessary.

**Results:**

Using somatic mitochondrial mutations as a marker for lineage tracing, it is possible to identify and follow tumor specific cell lineages. Using cycling temperature capillary electrophoresis (CTCE) a total of 8 tissues from 5 patients (4 primary tumors and 4 metastasis) containing clear mitochondrial markers of tumor lineages were selected. From these 8 tissues over 9,500 laser capture microdisection (LCM) samples were taken and analyzed, in a way that allows 3-D rendering of the observations.

**Conclusion:**

Using CTCE combined with LCM makes it possible to study the 3-D patterns formed by tumors and metastasis as they grow. These results clearly show that the majority of the volume occupied by a tumor is not composed of tumor derived cells. These cells are most likely recruited from the neighboring tissue.

## Background

Tumors are widely thought to be clonal lesions, originating from a single cell that grows and goes on to create a tumor mass (1–3). What are the patterns of growth and differentiation of this original cell lineage? To answer this fundamental question, the tracing of identifiable and specific markers in cell lineages is of interest as one of the methodologies for mapping the development. Somatic mitochondrial DNA (mtDNA) mutations are good candidates for markers of clonality and have been successfully used to study lineage tracing in normal human tissues (4–6). To implement such a strategy, small groups of cells from a histologic preparation (7) can be selected by laser capture micro dissection (LCM) for analysis. In normal tissue, histology and staining procedures combined with LCM can be employed in the selection of samples that contain cells from one tissue type only. In tumour tissue, additional difficulties make the lineage tracing methods less conclusive, because it can be difficult to distinguish tissue types within a tumor. While it is possible to recognize a tissue section as “not normal”, it is often impossible to determine which portions are tumor (8) and attempts to sample tumor tissue can therefore give biased results (1). Also, if tumors are indeed monoclonal, then there is only one lineage, but potentially of large size. In normal tissue, clones tend to be smaller, for example, one crypt in the human colon (6). This difference is probably the reason why in normal tissue even rare mutational events can be observed by repeated sampling of small amounts of tissue, because each sample originates from a distinct clonal unit. Attempts to detect gene inactivating mutations in cytochrome C oxydase in human adenomas on the other hand have been hampered by the small number of adenomas that actually develop such mutations (9). Each detected mutation usually covered a large portion of the total adenoma, as one would expect. Another issue with lineage tracing is the three-dimensional nature of tissue. Studying a thin slice cut from a tissue only gives a cross section of the real pattern of cell growth and cannot be relied upon to tell us about the spatial distribution. To understand the actual spatial spread, a three-dimensional (3-D) analysis is required and such an undertaking needs a multiple of the number of samples in the scan of a flat surface. Most methods that are capable of detecting and quantifying mtDNA mutations do not have the throughput and precision to satisfy this constraint (10).

**Figure 1:**
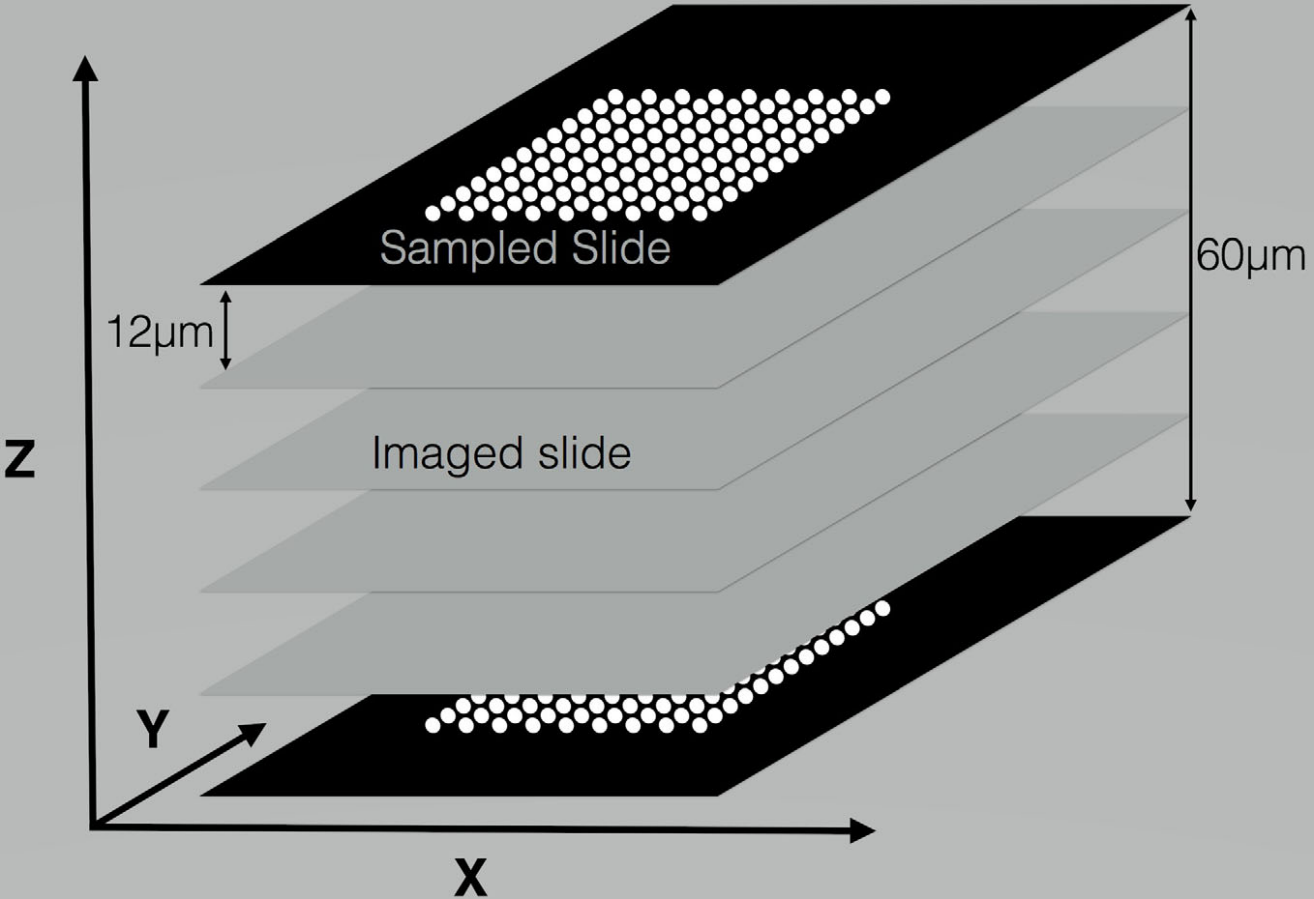
Schematic representation of sampling procedure. The sampled slides are placed on LCM membranes, and 96 samples are taken in the form of an 8 × 12 grid. One slide per 5 is sampled, and the ones in-between are placed on glass slides for imaging. The above schema has been repeated for a total of 70 slides of which *s* = 14 are sampled and 56 are only imaged. The total number of samples is 1,344.

In this paper, the authors develop a methodology to achieve this goal by adapting a process that has been shown to effectively detect and quantify mtDNA mutations in two dimensions, and extend it to the analysis of a three dimensional tumor mass. In the previous work (11), cycling temperature capillary electrophoresis (CTCE) was used to detect the presence of a mutation in the sample and then to quantify the relative amount of mutated to wild-type mtDNA. The method developed had the capacity to detect mutations in a large fraction of the total human mtDNA. We used the case of a Leydig cell tumor mass to demonstrate our methodology. The mutational analysis was performed on a large number of micro-anatomical samples forming a grid across a slice of tumor tissue. Since CTCE has good precision and high throughput, the 3-D analysis is feasible by taking consecutive thin slices from the tumor and taking within each slice samples on a standard grid formation at a roughly matching position on each successive slice of tissue. This makes it possible to align the slices and obtain a 3-D reconstruction. **Figure 1** shows the sampling stategy in a schematic manner.

## Materials and Methods

### Sample Description and Origin

Tumors were collected from 5 different patients, and have the following characteristics:

- Patient 1: Primary breast tumor, liver metastasis, lymph node metastasis;
- Patient 2: Primary bladder tumor, lymph node metastasis;
- Patient 3: Primary hepatocellular carcinoma;
- Patient 4: Primary colon cancer;
- Patient 5: Primary Leydig cell tumor [also described in (11)].

Samples from Patients 1, 2, and 4 were collected at the Norwegian Radiumhospital with informed consent. The sample from patient number 5 was collected at Bærum sykehus (Vestre Viken, Helse sør-øst, Norway). The sample from patient 3 was collected at the surgical department of the university hospital of Bologna (Italy). All samples were used with the informed consent according to local law and were anonymized prior to the analysis. According to the Norwegian law, “Technical and methodological development work that uses anonymized biological material” does not require approval from a research ethics committee (REC) (Web page, last access November 2016). Following surgical resections, all samples were snap frozen by immersion in liquid nitrogen within 10 min. They were then kept below –30°C until sectioning. No fixing or embedding was done before sectioning.

### DNA Extraction

Two consecutive 50 *μ*m thick slices were placed in a 1.5 ml centrifuge tube and DNA was extracted using the Mag-attract system by Qiagen (Hilden, Germany). The samples were incubated with Proteinaze K in lysis buffer for 3 h at 50°C and were then placed in a dedicated DNA extraction robot by Qiagen, specifically for the use of the Mag-attract system. This results in DNA at a concentration of approximately 50 ng/*μ*l.

### Tissue Sectioning

The tumor tissue is sampled by taking consecutive slices with the microtome and using LCM to sample a regular 8 × 12 grid on the slices (see **Figure 1**). Samples were mounted to a cryostat sample holder with a water-soluble glycols and resins matrix (Tissue-Tek O.C.T. Compound, Sakura, Finetek, USA) and placed in a cryotome (Leica CM1950) with room temperature of –20°C and knife temperature of –23°C. Small adjustments were made to those temperatures if it was necessary to improve cutting performance. 2 consecutive 50*μ*m slices were taken and placed in an Eppendorf (Eppendorf, Germany) tube. Next, 70 consecutive 12 *μ*m slices were taken. Every 5th slice (i.e. numbers 1, 6,… 66) was placed on a PET (Leica, Leica Microsystems, Wetzlar, Germany) membrane for LCM. All other cuts were placed on glass slides (SuperFrost by Thermo Scientific, Gerhard Menzel, Braunschweig, Germany) for imaging.

### Fixing and Staining

The membranes and glass slides with the 12 *μ*m tissue sections were dried at room temperature and fixed in pure methanol (Sigma-Aldrich) for 10 min, followed by subsequent air drying of the methanol residue. The tissue was stained for 30 min with Giemsa azur eosin methylene blue solution (Merck, Damstadt, Germany) (diluted 1/20 with H_2_O). The slides were then submerged for 30 s into a solution of 1% acetic acid in H_2_O (differentiation) and immediately rinsed in water. The stained tissue was air dried prior to imaging by microscope.

### Laser Capture Microdisection

A Leica LDM 6000 was used to take images of tissue sections mounted on membrane or glass slides. The software, Leica laser microdissection V6.7.1.3952, was used to control the microscope when taking pictures of selecting areas for laser capture microdissection and cutting. A hardware modification was made to the collection unit allowing for samples to be collected into two strips of 8 PCR caps (VWR, Oslo, Norway). The Leica software was used to design a standard sampling pattern of 96 circles, each with an area of about 25,000 *μ*m^2^, placed on an 8 × 12 grid (see **Figure 1**). The same grid was sampled on all membrane sections. Observation of the in-between samples placed on glass slides, allowed the grids to be placed in the position corresponding to the previous sampling. 20 *μ*l of a collection solution (1xThermopol buffer with Proteinase K (0.27 *μ*g*/μ*l) was added to each cap in the inverted strips. After cutting and collecting the selected areas by laser capture microdissection, the strips (with collection liquid and tissue) were mounted onto a 96-well PCR plate (Axygen, VWR, Oslo, Norway). The plate was briefly centrifuged and incubated at 56°C for 30 min. Deactivation of proteinase K was achieved by raising the temperature to 95°C for 1 min. One microliter of incubated solution was used as template for the first round PCR.

### PCR Conditions

#### First Round

Areas of mitochondrial DNA (mtDNA) that previously have been shown to contain many somatic mutations (12) were amplified with mitochondrial specific primers, designed to avoid amplification of homology regions in the nuclear DNA (NUMTs). Five sets of mitochondrial specific primer pairs were used, resulting in amplification product between 714 and 928 base pair in length (see **Table 1**). The PCR reaction mixture contained 0.1 *μ*l of extracted DNA, 0.8 mM dNTPs (0.2 mM of each dNTP) (VWR, Oslo, Norway), 1X Thermopol Buffer, 2 mM MgSO4, 0.075 unit Taq/*μ*l, 0.15 *μ*M of each forward, reverse and fluorescently labeled primer (Integrated DNA Technologies, Leuven, Belgium) in a total reaction volume of 10 *μ*l. The temperature cycling was performed in an Eppendorf Mastercycler ep gradient S (Eppendorf, Hamburg, Germany) with an initial denaturation 94°C for 240 s followed by cycling 38 time under the following conditions, denaturation at 94°C for 15 s, annealing for 40 s with temperature given in **Table 1** and elongation at 72°C for 150 s.

**Table 1:** First round mitochondrial specific primer pairs resulting in amplification product between 714 and 928 base pair in length.

#	Start (bp)	End (bp)	Length (Bp)	“Forward” primer (5’–3’)	“Reverse” primer (5’–3’)	Annealing Temperature (°C)
23	15,924	201	846	*AACCGGAGACGAAAACCTTTTTC	*CTTTAGTAGGTATGTTCGCCTGT	51
1	16,521	880	928	*CCATAAAGCCTAAATAGCCCACA	*CCAACCCTGGGGTTAGTATAGCT	54
10	6,917	7,671	754	*TGCTCTGAGCCCTAGGATTCATC	*TGAGGGCGTGATCATGAAAGGTG	55.5
16	10,852	11,566	714	*GCCTAATTATTAGCATCATCCCC	*ATGCCTCATAGGGATAGTACAAG	51
22	15,169	15,993	824	*GAGGGGCCACAGTAATTACAAAC	*TGGGTGCTAATGGTGGAGTTAAA	51
*=tail sequence (CGCCCGCCGCGCCCCGCG)						

#### Second Round

The primers used in the second round PCR are shown in **Table 2**. The template for second round PCR was 0.8 *μ*l of a 1:200 dilution (first round PCR in H2O). The templates were dispensed into 96-wells plates with a syringe dispenser (Hydra 96, Robbins Scientific, USA). To each well 10 *μ*l reaction mixture was added, consisting of 1xThermopol Reaction Buffer with 2 mM MgS04, 0.3 *μ*M forward primer, 0.15 *μ*M 1/2GC-tailed “reverse” primer, 0.15 *μ*M, 6-Carboxyfluorescein-GC-clamp primer, 500 *μ*M dNTP, 100 *μ*g Bovine Serum Albumine (Sigma-Aldrich, Oslo, Norway) and 0.75U Cloned Pfu DNA polymeraze. Plates were sealed with two strips of electrical tape (Clas Ohlson, Oslo, Norway). The temperature cycling was repeated 25 times; 94°C for 15 s, the annealing temperatures as given in **Table 2** held for 30 s and extension at 72°C for 60 s.

**Table 2:** Second round primer pairs used in the analysis.

#	Start (bp)	End (bp)	Template,fragment# from first PCR	“Forward” primer (5’–3’)	“Reverse” primer (5’–3’)	Annealing Temperature (°C)
1	16,569	25	1	*TGCATGGAGAGCTCCCGTGAGTGG	CCCCTTAAATAAGACATCACGAT	52
2	42	126	1	*ATTAACCACTCACGGGAGCTCTC	AGGATGAGGCAGGAATCAAAGAC	55
4	131	181	1	*CACCCTATGTCGCAGTATCTGTC	CACACTTTAGTAAGTATGTTCGC	55
6	483	513	1	*GGGGTTAGCAGCGGTGTGTGTGTG	TCCCACTCCCATACTACTAATCT	55
7	530	633	1	*TACCCAGCACACACACACCGCTG	CAAACCTATTTGTTTATGGGGTGA	55
8	673	705	1	*TTAGAGGGTGAACTCACTGGAAC	GGTTTGGTCCTAGCCTTTCTATT	58
82	7,031	7,134	10	*ACGACACGTACTACGTTGTAGCC	AATATGATAGTGAAATGGATTTT	52
84	7,340	7,416	10	*CTTTCTTCCCACAACACTTTCTC	TCTCAAATCATGAAAATTATTAAT	55
125	11,029	11,086	16	*TTAGGAGGGGGGTTGTTAGGGGGT	CATCCCTCTACTATTTTTTAACC	58
127	11,193	11,243	16	*ACCAGCCAGAACGCCTGAACGCA	GGTGTTGTGAGTGTAAATTAGTG	55
128	11,283	11,311	16	*TGTGCCTGCGTTCAGGCGTTCTGG	TAATCATATTTTATATCTTCTTC	60
130	11,437	11,492	16	*TTGACCCAGCGATGGGGGCTTCGA	GAGCCAACAACTTAATATGACTA	55
176	15,201	15,257	22	*AGAATCGTGTGAGGGTGGGACTGT	AGTAATTACAAACTTACTATCCG	60
177	15,274	15,377	22	*AGTAGACAGTCCCACCCTCACAC	GGTGATTTTATCGGAATGGGAGG	60
178	15,394	15,448	22	*CTAGGAATCACCTCCCATTCCGA	TAATGTCATTAAGGAGAGAAGGAA	55
181	15,761	15,864	22	*ACCTCCTCATTCTAACCTGAATC	CAGGCCCATTTGAGTATTTTGTTT	55
184	16,080	16,130	23	*CAAGTATTGACTCACCCATCAAC	ACAGGTGGTCAAGTATTTATGGTA	57
185	16,170	16,272	23	*GTGGGTGAGGGGTGGCTTTGGAGT	CCAATCCACATCAAAACCCCCTC	56
187	16,263	16,366	23	*AACTGCAACTCCAAAGCCACCCC	CCCTATCTGAGGGGGGTCATCCAT	58
				*=tailsequence(CCCGCCGCCCCCGCCCGGG)		
				GC-Clamp=(6FAM-CGCCCGCCGCGCCCCGCGCCCGTCCCGCCGCCCCCGCCCGGG)		

### Cycling Temperature Capillary Electrophoresis

A CTCE analysis was performed for the selected fragments as previously described. A 96-capillary DNA analyzer (MegaBACE 1000) was used to analyze 6-carboxyfluorescein labeled PCR products. Mutant PCR amplicons were separated from the wild type by cycling the temperature in the vicinity of the capillaries. The cycling temperature was based on the theoretical melting temperature, for each fragment, calculated by Poland’s algorithm (13) in the implementation described by Steger (13, 14). The separation temperature proposed by the algorithms was adjusted based on the urea concentration in the matrix. The instrument was modified to allow for elevated temperature cycling (15, 16). Temperature cycling was programmed in the macro.ini file used by the Instrument Control Manager (ICM) software package (GE Healthcare Life Sciences, Pittsburgh, PA, USA). The injection and running electric fields were as given for the first round amplicons.

### Limitations

In all tissues, even though the measurement procedure is complex, valid observations were obtained in more than 80% of the samples. The most likely source of failure is the LCM system, which relies on gravity to transfer the cut sample into the well. Due to the small size and weight of the sample, as well as the static electricity present on the membrane, samples sometime do not fall. Also, a high standard of quality was kept, and only samples in which a mutant fraction could be cleanly measured in the CTCE run, with a precision below 3–5%, were retained. The automated analysis of the electropherogram requires a very high quality signal as well as an internal standard.

The ability to make a three dimensional analysis of cell lineages in tumor depends on the quality of the tissue. Both primary tumors of the breast and prostate were low quality tissues. They were small and poorly conserved and it was impossible to take the 70 consecutive high-quality slices. A smaller number of samples *s* was therefore taken for the purpose of understanding the distribution of the mutations. With the resulting data, the 3-D plotting was still available for viewing. Since the primary goal of the paper consists in proving that 3-D lineage tracing is feasible, rather than in developing a methodology that can be used routinely by cancer researchers, we did not pursue the transfer of technology aspects.

### Data Analysis

The outcome of the analysis is the measured mutant fraction in the samples. Because each spot is associated with three spatial coordinates (*x*, *y*, *z*) arranged in a 8 × 12 × *s* grid, understanding these results requires the information to be analyzed and visualized in three dimensions. The hypothesis is made that the mutation marks a cell lineage within the tissue. For each sample, the depth *z* within the tissue is known by the slice number. To the best of our ability, the sampling grids within the slices are arranged in the same position (*x*, *y*) on each sampled slice, which allows us to assign a cartesian coordinate (*x*, *y*, *z*) to each sample taken. Also, the volume of the sample is constant, a disc of 25,000 *μ*m^2^ with a height of 12 *μ*m.

In analysing the measured mutant fractions, marginal (1-D) methods such as histograms and curves, heatmaps (2-D), and 3-D tools were used.

## Results

**Table 3** summarizes some aspects of the tissues measured for this paper. A 3-D reconstruction of the mutant fractions has been possible for six tissues. Of these, two tissues have two markers each, for a total of 8 three dimensional reconstructions. Not every sample gives valid information, due to the morphology of the tissue, PCR failure, or difficulties in the interpretation of the CTCE signal. The total number of samples taken, as well as the number of successful ones are shown in **Table 3**.

**Table 3:** Summary of samples within each tumor or metastasis.

Patient	Tissue	3-D	Markers	Sample Taken	Measured Samples
1	Primary Breast tumor Lymph node metastasis Liver Metastasis	no yes yes	1 1 1	672 1,344 1,344	524 571 708
2	Primary Bladder cancer Lymph node metastasis	no yes	2 2	288 1,344	287, 229 986, 611
3	hepatocellular carcinoma	yes	1	1,344	956
4	Colon Cancer	yes	1	1,344	598
5	Leydig cell tumor	yes	2	1,344	1,728, 1,824

For each patient, the markers analyzed are the same across the tumor tissue. In some tissues, due to their states or size, it was impossible to establish a 3-D reconstruction. Such a reconstruction was only established when multiple samples could be taken consecutively. The number of samples taken is the number of micro anatomical pieces taken using LCM. The measured samples are those for which a mutant fraction could be successfully calculated. In the cases with two markers, the number of samples for each marker are separated by a comma.

**Figure 2:**
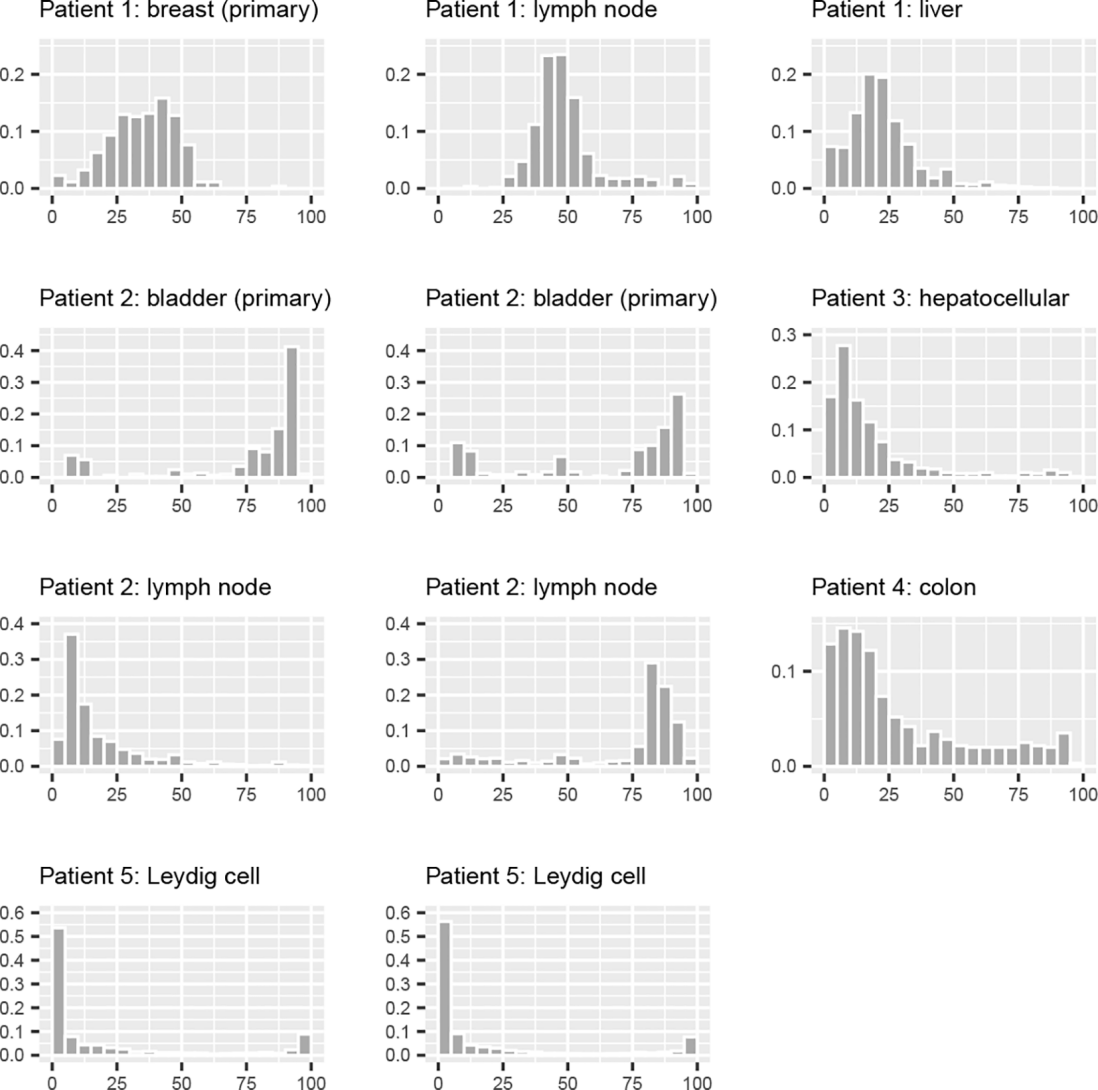
This plot shows the histograms of the mutant fractions for each tumor. The horizontal axis is binned into 0–5%, 5–10%, and so on. The vertical bars show the portion of the measured mutant fractions falling into each bin. The first row shows the primary tumor of patient 1 together with the two metastases, all carrying the same mutation. The hepatocellular carcinoma and the colon cancer, are from patients with only one tissue, each with one marker mutation. Patient 2 has a primary bladder tumor with two different mutations, both of which are found in the lymph node metastasis. The Leydig cell tumor carries two mutations with remarkably similar distributions.

**Figure 2** shows the histograms of the mutant fractions expressed as percentages between 0 and 100 for the 11 mutations sampled within the five tissues. Patient 1 and 2 have primary tumors as well as metastases and the fact that the same mutations are found in both primary tumor and metastasis gives strong support to the hypothesis that these are markers for the tumor cells.

The different shapes of the distributions of the mutant fractions have to be interpreted in light of the mutational process in mitochondria, which differs in important aspects from nuclear DNA. First of all, mtDNA is present in multiple copies in all cells and this makes the passing of a mutation during cell doubling uncertain. If all or none of the mtDNA copies carry the mutation, the daughter cells will be of the same type. This makes 0% (wild type) and 100% (homoplasmic mutant) fixed states of the process. Everything inbetween will over sufficiently many cell divisions converge to either of these two fixed states, leading to a U-shaped distribution. Both markers in the Leydig cell tumor are close to this state, as are the two markers for the primary bladder cancer. Others are somewhere in between. It has been shown that in a particular model of the mutational process, the convergence from a cell with few mutant copies to homoplasmic mutant state happens surprisingly quickly (17). Note that the multiplicity of the copies accelerates this process, because in each cell doubling, the number of mutant copies doubles and many of them may end up in the same daughter cell. Keeping this in mind, a fraction of the samples with 0% mutant fraction belong to the tumor cell lineage, the mutation simply has been washed out during the progression of the lineage. On the other hand, if a sample has a high concentration of mutations, then the surrounding samples both within the same slice and on neighboring slices are expected to have a high concentration, if descendent or ascendent cells from the sample are present.

A majority of the samples carry the mutational marker at a low rate. For two of the three tissues with two marker mutations, both have similar distributions. This together with the 3-D image suggests that the mutations are found together in the same samples, thus independently marking the same lineage. In the lymph node metastases, the two marker mutations have very different distributions. This is probably due to the seeding of the metastasis with cells having different mutant fractions in the two markers.

**Figure 3:**
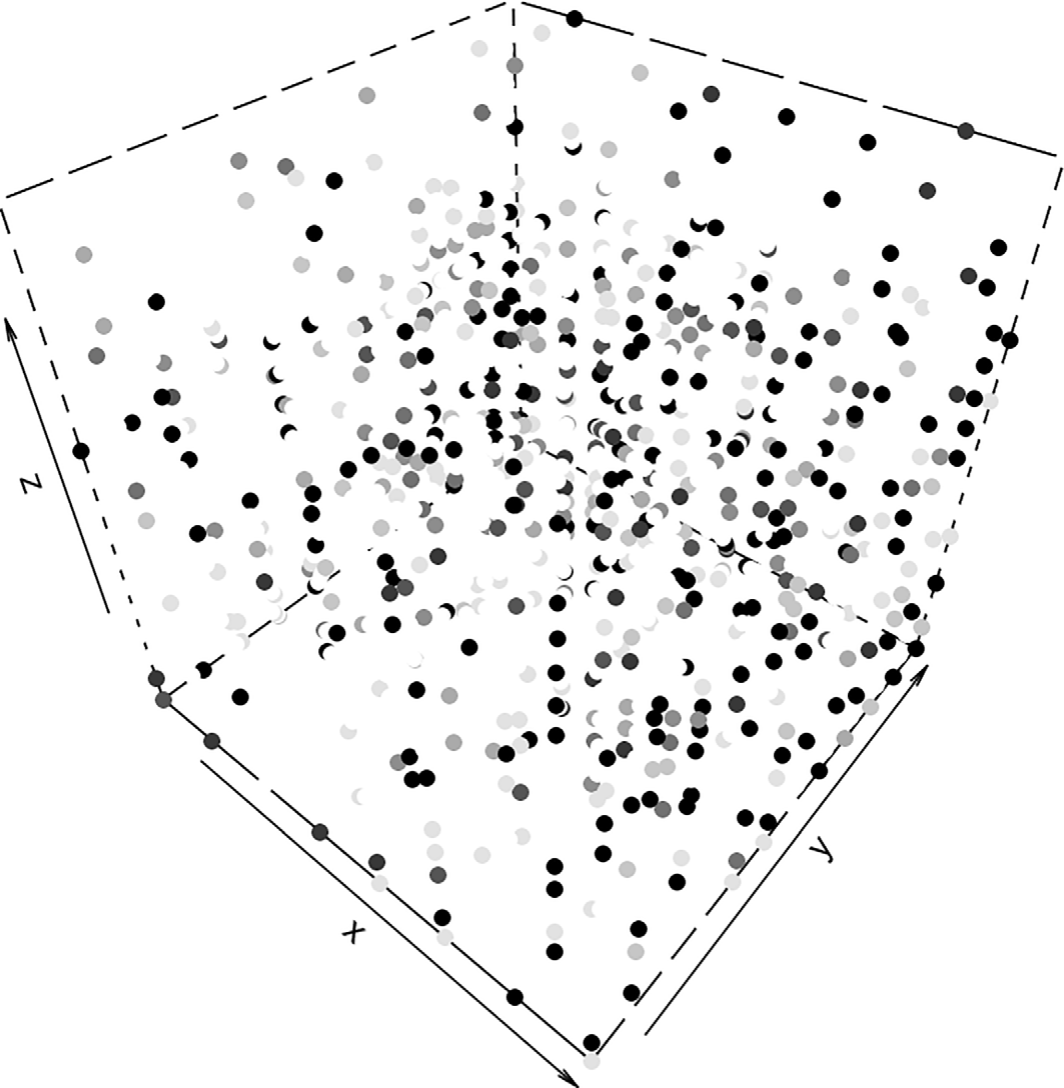
A grey-level coded presentation of the mutant fractions at 8 × 12 sites on *s* = 18 slices. The total number of samples is 1,728. The data are for the first marker of the Leydig cell tumor. The darkness of the grey scale indicates the mutant fraction (white = 0%, black = 100%). One can discern that at the center of the cubic structure, the mutational marker seems to be more prevalent and more dense. But high mutant fractions occur at sites throughout the tissue.

Since the data are sampled in a 3-D arrangement of spots, a 3-D plot of the mutant fractions is a natural form of visualization. **Figure 3** shows such a plot with each point in the cube colored according to the value of the mutant fraction. Points with zero fraction are intentionally left white in order to allow for transparency when judging the spatial arrangement of the points with positive fractions. This and all the subsequent figures are based on the first mutation in the Leydig cell tumor.

**Figure 4:**
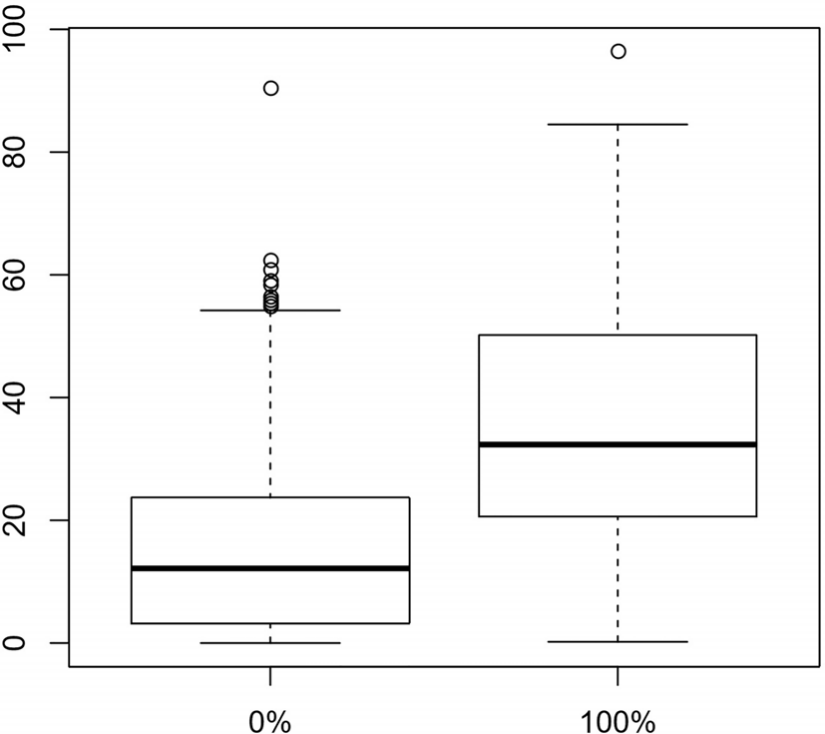
Boxplots of the mean mutant fractions for neighbors in the *x*, *y* and *z* directions of spots with 0 and 100% mutant fractions show a significant difference. Spots carrying the mutation have neighbors with higher mutant fractions. Wilcoxon’s two-sample test gives a p-value of 2.2 × 10^-16^ in favor of unequal local averages around the two types of central sites.

A sizeable fraction of the volume does not carry the mutation. There is a layer in the middle of the tissue with a higher fraction than the rest, and there is a spatial connection between the areas carrying the mutation. To test this non-randomness of the spatial distribution we computed the average mutant fraction of the samples that are neighbors in any direction of spots with a 100% mutant fraction and compared these averages with those obtained in the neighborhood of spots with 0% mutant fraction. Boxplots of these average mutant fractions are shown in **Figure 4**. There is a clear proof of a clustering effect of the spots with higher mutant fraction. If we consider larger neighborhoods, the difference between the two locally averaged mutant fraction drops off. The cells carrying the marker percolate through the tumor tissue, but do not permeate it.

**Figure 5:**
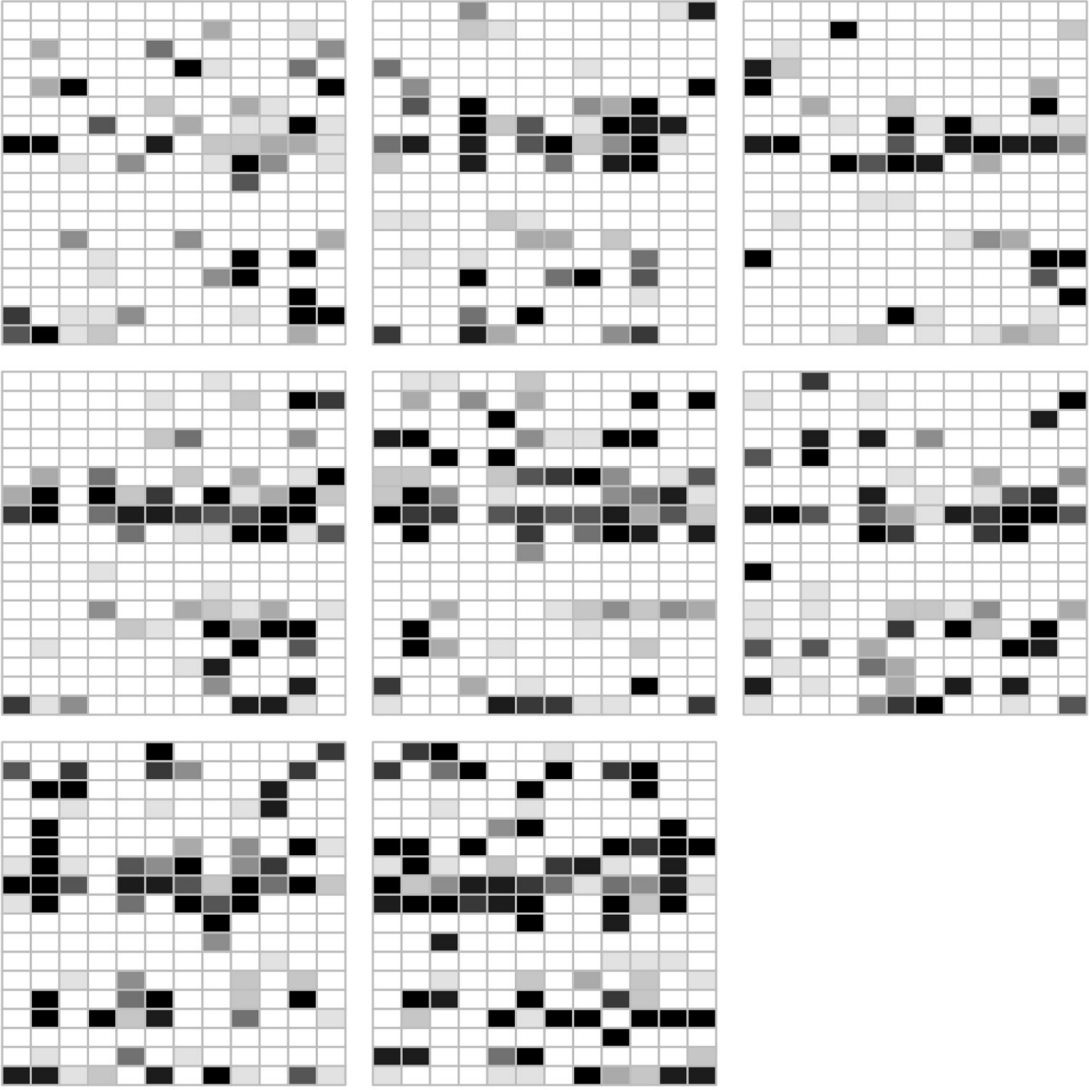
For each (*y*, *z*) slice with a fixed *x* coordinate these heatmaps show the mutant fraction in the 12 × 18 samples for the first maker in the Leydig cell tumor. To reconstruct the 3-D plot, the slices would have to be stacked. Spots in neighboring heatmaps at the same site are direct neighbors of each other in the direction of the *x*-axis.

Alternatives to the 3-D plots that allow a closer inspection of particular regions have to be based on 2-D visualization tools. **Figure 5** shows the example of the Leydig cell tumor in which we analyze the slices in (*y*, *z*) for fixed values of the x-coordinate. Each slice contains 8 × 12 samples and there are 8 such slices. For values of *z* near the middle, all of the 8 plots show a horizontal arrangement of high mutant fractions. This shows that the (*x*, *y*) slice for a fixed z-coordinate cutting close to the center of the cubic structure has a high mutant fraction throughout the slice. **Figure 5** also shows that the patterns are remarkably similar across all slices and consistent with a lineage dependence.

**Figure 6:**
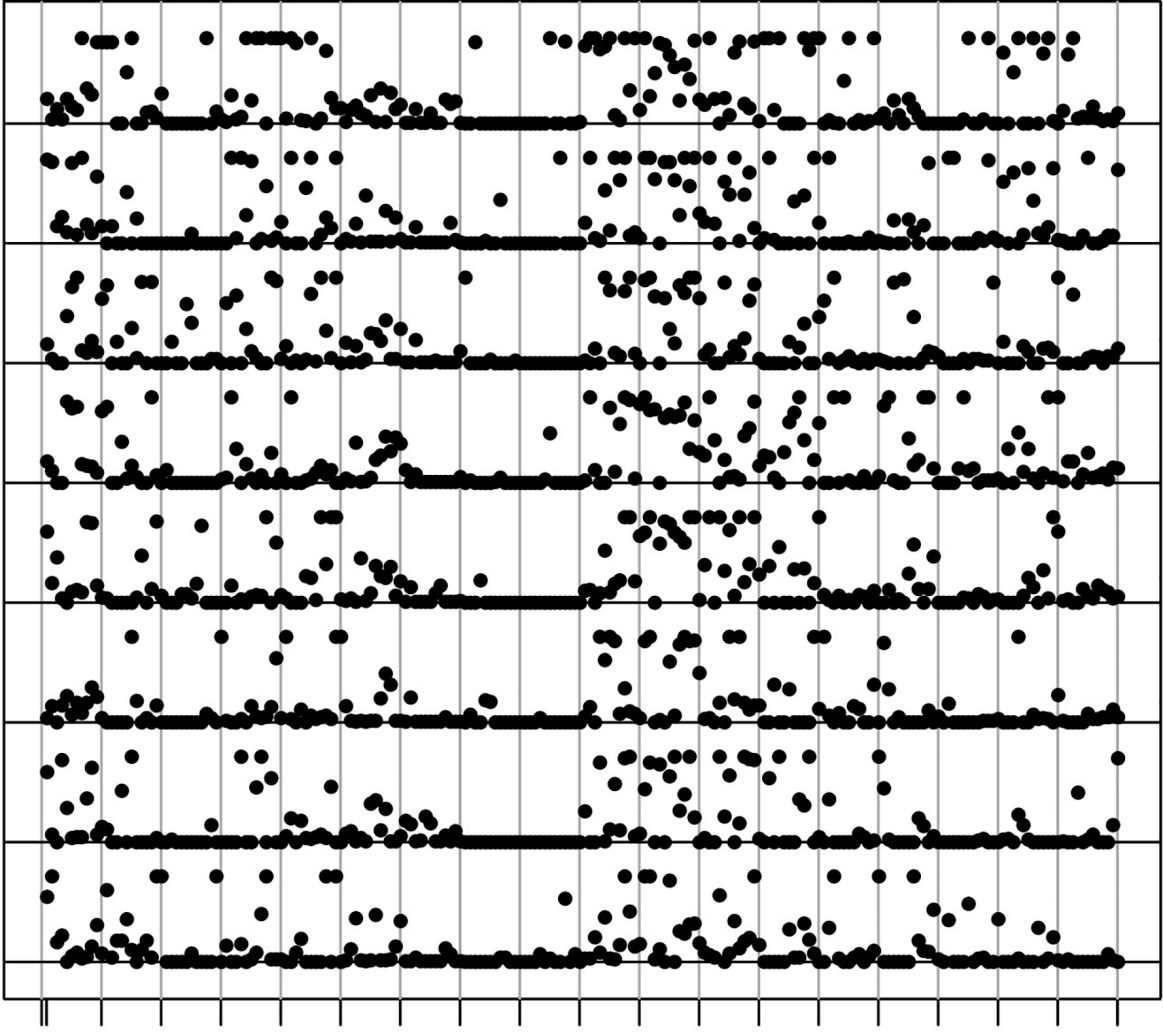
The same data as in the parallel heatmaps is shown here in the form of stacked curves. Each of the curves shows the mutant fractions for a slice with a fixed x-coordinate slice. The horizontal axis refers to the sites in the (*y*, *z*) slices. The vertical lines and the tick marks partition the axis in 18 intervals, with each interval having a fixed z-coordinate. Here, both a direct comparison of samples with fixed x-coordinate as well as the comparison across the different x-values is encouraged.

A second example of stacked plots is shown in **Figure 6**. Here, the slices shown as square plots in **Figure 5** are arranged as 8 curves along a single axis. The 12 × 18 mutant fractions are arranged in a row. These curves make the strong correlations across the whole cube even clearer. The cell lineage contains older and more recent parts and we can only speculate which is which. A cylindrical slice along the x-axis between 100 and 150 has many samples with high mutant fractions. Next to it is a region with lower levels of the marker, but then high concentrations of the marker emerge again near the *x* = 0 edge of the cube.

**Figure 7:**
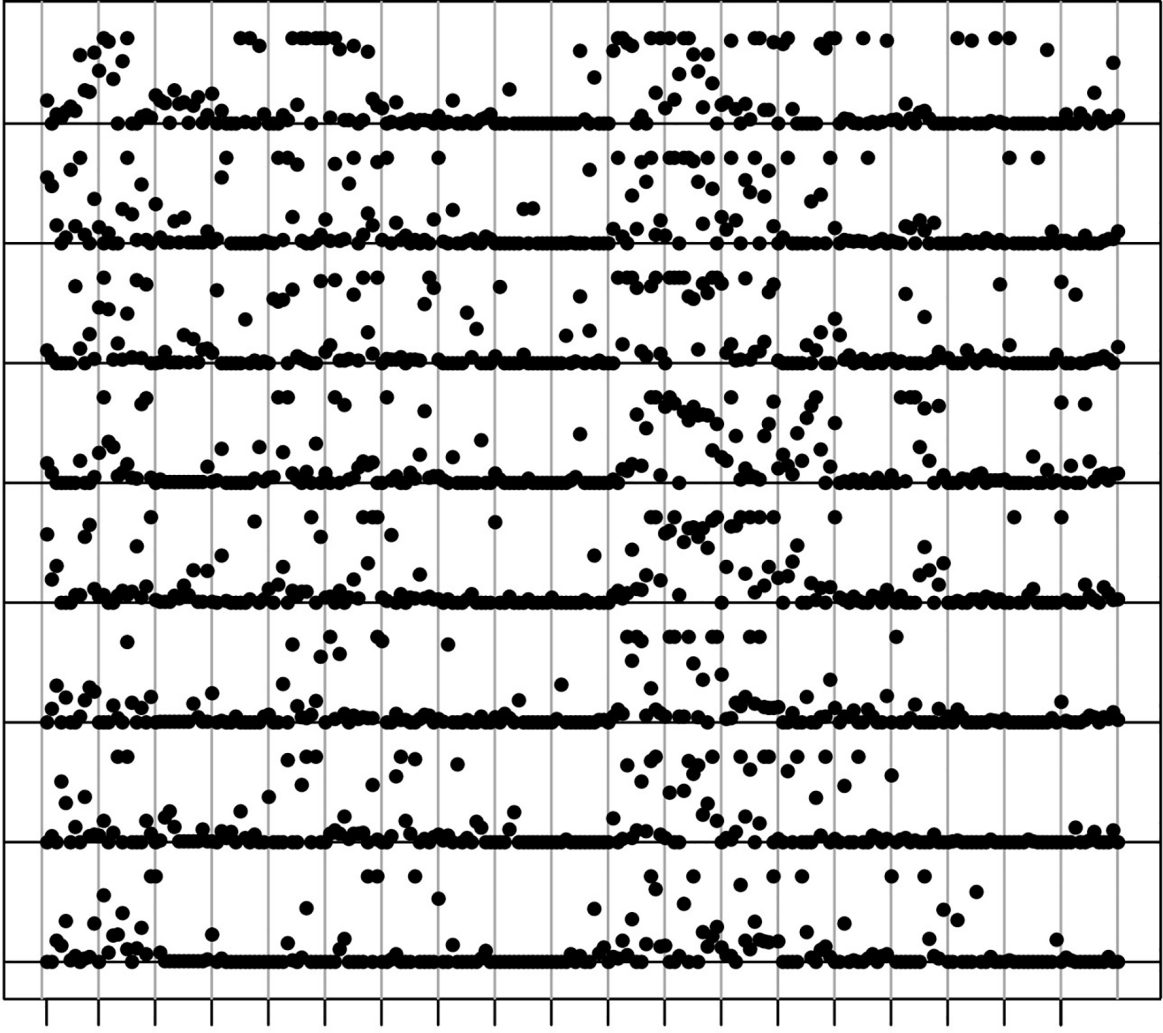
This is the stacked curves plot for the second mutational marker found in the Leydig cell tumor. There is an additional *z* coordinate compared to the first marker, which results in 12 × 19 = 228 samples for each of the 8 slices.

Not shown in the previous two plots is the strong similarity observed between marker 1 and marker 2 of the Leydig cell tumor. **Figure 7** shows the second marker in the form of stacked curves. A comparison with the previous figure shows a remarkable overall similarity. This observation supports the hypothesis that there is one cell lineage that carries both markers. By studying the 3-D reconstruction of the Leydig cell tumor, it can also be noted that the majority of the volume studied is free of both mutations. In fact, we never observed a tumor that was majority mutation carrying.

## Discussion

We examined several surgically removed tumor tissues using mtDNA mutations as markers for cell lineages. A 3-D scan of tumor volumes was made possible through the use of slices together with samples arranged on a grid in each slice. The results showed that the grid-based sampling provides a good basis for a 3-D analysis and allows for an unbiased selection of the samples. The presence of an internal standard assures that the mutation being mapped is the same in each sample, because the probability of two lineages carrying the same mutation is estimated to be below 1/700 (11). Other papers estimate this probability to be even smaller (4, 5).

**Figure 8:**
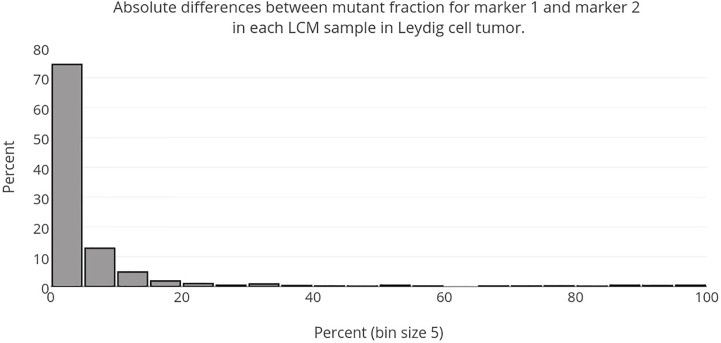
The absolute value of the difference between the mutant fraction of marker 1 and marker 2 is taken for each LCM sample of the Leydig cell tumor. The histogram is then made with the resulting values. As can be seen, the majority of the values cluster around a difference of 0, which demonstrates the strong similarity between the two observed mutant fractions across the whole volume.

The distributions of the two markers observed in the example of the Leydig cell cancer are remarkably similar (see **Figures 2**, **6**, **7**). The 3-D reconstruction confirms the strong similarity and shows that areas carrying one mutation also carry the other. A further confirmation is shown in **Figure 8**, which is a histogram of the difference is the mutant fractions. In the vast majority of the spots, the two mutant fractions are within 5% of each other.

Standard theory of cancer development suggests that metastases are seeded by one, or a few cells (18–22). In the case of the breast tumor we analyzed, two metastases were found to carry the same mutation as the primary tumor. This strongly supports the hypothesis that the lineage marked by the mutation is indeed a tumor lineage. Furthermore, if it is true that one or very few cells were at the origin of the metastasis, then the only tumor lineage present in the metastasis, is the one marked by the mutation. In the primary tumor, it cannot be excluded that other tumor lineages exist and that the 3-D analysis shows the distribution of one tumor cell lineage.

A part of the volume of the tumor which does not contain any mtDNA mutation can nevertheless contain tumor cells (as discussed in Section 3). Another part of this volume, however, is likely to be tumor free, having been recruited from normal cell tissue. We hypothesize that a sizeable part of the tumor is not composed of tumor-derived cells. The tumor cells are scattered in small clusters that are sometimes connected. The 3-D images resemble a percolation pattern which can be interpreted as a diffusion of the tumor cells through the tissue occasionally followed by small local growths. The presence of these cells, however, disrupt the local tissue to assume a “disorganized” morphology.

## Conclusion

In the three cases where two markers were analyzed independently, the results are consistent and, in the example of the Leydig cell cancer, confirm the precision of the methodology. The analysis described here is not limited to mtDNA mutations. With further development it can be extended to mutations on nuclear genes.

Three-dimensional reconstructions of tumor cell lineages is consistent with the hypothesis that a sizeable part of the tumor volume is not composed of tumor-derived cells. The investigation of metastases yields additional evidence in favor of this view. The observations described here support two models of tumor growth. A part of the growth of a tumor or a metastasis is through recruitment of cells from normal tissue; or through the spread and diffusion of tumor cells through the host tissue. Such a process of tumor growth creates the conditions leading to the observed abnormal morphology. These models are further supported by the similarity of the 3-D reconstructions in all the analyzed cases.

## Data Availability Statement

The datasets generated for this study are available on request to the corresponding author.

## Ethics Statement

Ethical review and approval were not required for the study on human participants in accordance with the local legislation and institutional requirements. Written informed consent for participation was not required for this study in accordance with the national legislation and the institutional requirements.

## Author Contributions

PR and PE planned and performed the experiments. SM performed all statistical analysis. PE, SM, and PR worked together to write the manuscript. WT provided background context for the study, contributed to writing the manuscript, and helped in interpreting the results. All authors contributed to the article and approved the submitted version.

## Funding

Research has been performed with the operational funds of the Chair of Applied Statistics (STAP), from EPFL.

## Conflict of Interest

The authors declare that the research was conducted in the absence of any commercial or financial relationships that could be construed as a potential conflict of interest.
